# TIPIT: A randomised controlled trial of thyroxine in preterm infants under 28 weeks gestation: Magnetic Resonance Imaging and Magnetic Resonance Angiography protocol

**DOI:** 10.1186/1471-2431-8-26

**Published:** 2008-06-30

**Authors:** Sze M Ng, Mark A Turner, Carrol Gamble, Mohammed Didi, Suresh Victor, Christina Malamateniou, Laura M Parkes, Anna Tietze, Lloyd Gregory, Vanessa Sluming, Laurence Abernethy, Alan M Weindling

**Affiliations:** 1 School of Reproductive and Developmental Medicine, University of Liverpool, Liverpool, UK; 2 Centre for Medical Statistics and Health Evaluation, University of Liverpool, Liverpool, UK; 3 Department of Endocrinology, Royal Liverpool Children's Hospital, Liverpool, UK; 4 Maternal and Fetal Health Research Group, School of Clinical and Laboratory Sciences, Faculty of Medical and Human Sciences, University of Manchester, UK; 5 School of Health Sciences, University of Liverpool, Liverpool, UK; 6 Magnetic Resonance Imaging and Analysis Research Center (MARIARC), University of Liverpool, Liverpool, UK; 7 Wellcome Trust Clinical Research Facility, Manchester, UK; 8 Department of Radiology, Royal Liverpool Children's Hospital, Liverpool, UK

## Abstract

**Background:**

Infants born at extreme prematurity are at high risk of developmental disability. A major risk factor for disability is having a low level of thyroid hormone described as hypothyroxinaemia, which is recognised to be a frequent phenomenon in these infants. Derangements of critical thyroid function during the sensitive window in prematurity when early development occurs, may have a range of long term effects for brain development. Further research in preterm infants using neuroimaging techniques will increase our understanding of the specificity of the effects of hypothyroxinaemia on the developing foetal brain. This is an explanatory double blinded randomised controlled trial which is aimed to assess the effect of thyroid hormone supplementation on brain size, key brain structures, extent of myelination, white matter integrity and vessel morphology, somatic growth and the hypothalamic-pituitary-adrenal axis.

**Methods:**

The study is a multi-centred double blinded randomised controlled trial of thyroid hormone supplementation in babies born below 28 weeks' gestation. All infants will receive either levothyroxine or placebo until 32 weeks corrected gestational age. The primary outcomes will be width of the sub-arachnoid space measured using cranial ultrasound and head circumference at 36 weeks corrected gestational age. The secondary outcomes will be thyroid hormone concentrations, the hypothalamic pituitary axis status and auxological data between birth and expected date of delivery; thyroid gland volume, brain size, volumes of key brain structures, extent of myelination and brain vessel morphology at expected date of delivery and markers of morbidity which include duration of mechanical ventilation and/or oxygen requirement and chronic lung disease.

Trial registration

Current Controlled Trials ISRCTN89493983

## Background

Extremely premature infants are at high risk of disrupted brain development with consequent acquired damage, especially to the white matter because of multiple factors in an adverse environment. Adverse conditions are thought to include hypoxic ischaemia, free radical injury i.e. factors related to their premature birth because of low concentrations of antioxidants, undernutrition and sepsis[1]. At untimely extreme premature delivery (24 – 28 weeks of gestation), brain development is at a critical stage because neuronal migration has only just completed and synaptogenesis is occurring: therefore the cellular milieu is critical[2].

Brain magnetic resonance imaging (MRI) studies of survivors of low birth weight who have subtle cognitive abnormalities have shown diminished volumes of the caudate nucleus and hippocampus. Diminished volumes of the caudate nucleus and hippocampus were associated with lower IQ, learning disorder and attention deficit[3,4]. Recent brain MRI studies of preterm infants also show that quantitative cerebral structural abnormalities are related to the degree of immaturity at birth and are followed by abnormal neurodevelopmental outcome[5]. Preterm infants assessed at term show reduced myelination when compared to infants born at term[5,6]

Only 70% of infants born before 26 weeks' gestation survive until expected date of delivery[7]. Of the survivors at least half have significant neurocognitive disabilities[8,9]. Many factors are associated with an increased risk of neurocognitive impairment. Brain growth following birth is a major determinant of neurocognitive outcome in these infants and poor brain growth is recognised as a dominant determinant of neurocognitive outcome[10]. Brain growth at the expected date of delivery in preterm infants are key predictors of long-term brain growth and intelligence quotient (IQ) score[10]. This implies that postnatal head growth is a more important determinant of IQ than intrauterine growth.

Observational studies have found that low thyroid hormone levels in the first few weeks of life in preterm infants are associated with poor neurodevelopmental outcome. (see Table 1). These associations persist after adjustment for confounders and suggest that hypothyroxinemia during the critical window period for brain development which occurs following birth at extreme prematurity has important effects on neurodevelopment. However, at present it is unclear whether poor neurodevelopment is caused by hypothyroxinaemia or whether the hypothyroxinaemia is a consequence of other factors that also cause poor neurodevelopmental outcome and simply an associated abnormality.

**Table 1:** Hypothyroxinaemia in preterm infants is linked with poor neurodevelopmental outcome

Study	Participants	Outcome
Reuss et al [57]	463 infants born below 33 weeks' gestation	4-fold increased risk of disabling cerebral palsy at 2 years of age associated with hypothyroxinaemia
Den Ouden et al [58]	563 infants born below 32 weeks' gestation	Higher incidence of neurological dysfunction at 5 and 9 years of age associated with hypothyroxinaemia
Leviton et al [36]	1414 infants weighing 500 to 1500 g	Double the risk of cerebral white matter changes associated with hypothyroxinaemia
Paul et al [59]	343 infants weighing less than 1500 grams	Increased mortality and incidence of intraventricular haemorrhage associated with hypothyroxinaemia
Lucas et al [60]	279 infants born below 36 weeks' gestation	Reduction in IQ at 8 years of age associated with low T3
Meijer et al[61]	563 infants born below 32 weeks' gestation and/or birthweight less than 1500 g	Negative score on the three milestones of development at 2 years of age associated with hypothyroxinaemia

There is prime facie evidence that thyroid hormones directly contribute to brain development which leads us to suspect that hypothyroxinaemia among preterm infants has adverse effects on brain development. Animal studies have shown that a deficiency of thyroxine results in impaired neurological pathways during early neurogenesis which results in abnormalities of the cytoarchitecture of specific brain structures such as the hippocampus, temporal and sensorimotor cortex [11-15]. Eayrs and colleagues have shown in early studies[16,17] that perinatal hypothyroidism in rats alter the density and size of neurones and fibre density in specific brain regions and the orientation of cortical layers. Berbel et al[18,19] also described effects on spine density of pyramidal neurones in the cerebral cortex, organisation of callosal connection and maturation of dendrite connections as a result of hypothyroidism. Thyroid hormones are also demonstrated to increase proliferation of cerebellar granule cells as well as affect apoptosis which is important in suppression of proliferation and downgrading of these cells[20,21]. Thyroid hormones are essential for normal axonal myelination and for normal interhemispheric commisure development[22,23]. Thyroid hormones are also important in regulating transcription of specific genes expressed in the developing brain[24]. This suggests an important role for thyroid hormone in the complex developing brain throughout gestation.

While neurocognitive outcome in childhood is the long-term concern, previous work has demonstrated that the size of key brain structures at expected date of delivery is a predictor of subsequent development and is a more immediate way to evaluate strategies that aim to enhance brain growth [9,33]. This is because the size of brain structures reflect the extent of myelination. Myelination is the process by which nerve fibres are coated with thin layers of lipid called myelin. Myelin acts as electrical insulation and increases the efficiency of nerve fibres. In children with a congenital lack of thyroid hormone (congenital hypothyroidism) there is a lack of cerebral myelination[25,26]. Thyroid hormones are therefore a factor in the regulation of the extent of myelination during gestation and after birth. Furthermore, TSH receptors have been found in early human foetal brain and human astrocytes in culture, and they are thought to mediate extrathyroidal cyclic AMP independent biological effects of TSH and stimulation of the type 2 deiodinases in astroglial cells.[27]

At present, there is a paucity of published data concerning the relationship between thyroid hormone status and brain development in extreme preterm infants. Previous work has demonstrated that the size of key brain structures and brain volume at term is a key predictor of subsequent development[2,6,9,28]. Current evidence also shows that hypothyroxinemia is common in extreme prematurity [29-33]. Derangements of critical thyroid function during the sensitive window in prematurity when early development occurs could have a range of long term effects for brain development.

A randomised controlled trial of levothyroxine supplementation by van Wassanaer [34,35] enrolled 200 infants under 30 weeks gestation showed no overall effect of levothyroxine supplementation on medium- or long-term outcomes. However, post hoc subgroup analysis showed an improved Bayley MDI and PDI at 2 years and 5.5 years of follow up among infants born below 27 weeks' gestation and a better motor outcome in those born below 28 weeks' gestation in the treatment arm. Paradoxically, the reverse was true for those infants born at 29 weeks' gestation and above which had a worse Bayley MDI and PDI developmental score. However, the sample size for this sub-set of infants with gestational age under 27 weeks who benefited from the intervention was small with 19 infants in the treatment arm and 27 in the placebo arm. Several reasons may explain this gestation dependent positive effect of thyroxine in infants born below 28 weeks' gestation. Firstly, FT4 concentration in the very immature infants may be too low to ensure normal brain development, of which thyroid dependent maturation in the brain takes place in the window period before 30 weeks. It is also possible that thyroid hormones are important in its role of repairing ischaemic damage of the brain which commonly occurs in extreme prematurity[36].

This study is an explanatory randomised controlled trial of levothyroxine vs placebo in infants born below 28 weeks' gestation to determine the effects on brain volume, and development, the hypothalamic-pituitary-adrenal (HPA) axis and somatic growth (see TIPIT protocol[37]).

This trial will provide the opportunity to assess whether supplementation with a thyroid hormone during development up until 32 weeks gestation affects the extent of myelination because we are undertaking detailed cranial MRI in a subset of trial participants. An MRI technique called Diffusion Tensor Imaging will assess the structural integrity of the white matter[38,39]. The anisotropy of water diffusion will be measured which is dependent on the degree of myelination. In addition, we aim to qualitatively and quantitatively evaluate the effect of thyroid hormones on cerebro-vascular morphology, including vessel size (using measures of diameter), architecture (using measures of tortuosity) and vessel density in extremely premature infants and to understand more about the interdependence of vessel and brain development and their relationship to prematurity. In addition, this trial will determine whether the effects of levothyroxine on the regulation of brain development are clinically relevant.

Important outcomes that will be assessed with cranial MRI are the extent of white matter myelination and integrity, vessel morphology and the volumes of key brain structures at term equivalent which will be detailed in this protocol.

All infants recruited to the TIPIT study[37] will be consented separately to have cranial MRI scans at term equivalent. This protocol describes the study we will perform on the infants that have cranial MRI scans.

### Hypothesis

We speculate that a deficiency of thyroxine results in impaired neurological pathways during early neurogenesis which results in abnormalities in myelination and alters the cytoarchitecture of specific brain structures such as the hippocampus, caudate nucleus and thalamic nuclei. The study hypothesis is that supplementation with levothyroxine is associated with significant differences in markers of myelination and brain architecture on cranial MRI.

### Objectives

To measure markers of myelination and brain architecture on cranial MRI among extreme preterm infants (< 28 weeks gestation) recruited to an explanatory randomised controlled trial[37] to determine the effect of thyroid replacement therapy on:

• whole brain volume

• specific brain structures volumes- caudate, hippocampus, thalamus

• pituitary height

• brain vessel morphology

• white matter structural integrity

## Methods

### Design of TIPIT study

A randomised placebo controlled trial of levothyroxine postnatally until 32 weeks' corrected gestational age (CGA) in infants born below 28 weeks' gestation, with blinding of parents, care providers and outcome assessors. Infants will be enrolled as soon as possible within five days of life.

#### Study population

All infants must fulfill the inclusion criteria.

Inclusion criteria:

a) Infants with gestational age below 28 weeks at birth

b) Recruitment within five days of birth

c) Single or multiple births

Exclusion criteria:

a) Infants born to mother with known thyroid disease or on antithyroid medications during pregnancy

b) Infants born to mother who are on amiodarone during pregnancy

c) Infants diagnosed with major congenital or chromosomal abnormalities known to affect thyroid function or brain development

d) Maternal death during or within 5 days after childbirth.

#### Study Medication

We will use two forms of the active medication: intravenous levothyroxine (tradename: Levothyroid) and oral levothyroxine solution (tradename: Evotrox) with corresponding placebos during the study period.

In the initial phase, infants will receive either intravenous Levothyroid or placebo (5% dextrose) within the first 5 days after birth. The treatment regime will follow the dosage guidelines determined by previous studies at 8 mcg/kg birthweight/day single daily dose.

In the next phase, once enteral feeds are fully established, oral solution Evotrox or placebo (carrier solution without active drug) will be given at 8 mcg/kg birthweight/day single daily dose until the baby reaches 32 weeks CGA.

#### Overview of assessments

• Thyroid hormone levels and the HPA axis will be measured at baseline, day 14, day 21, day 28 and 36 weeks (corrected age).

• Growth and auxology measurements will be measured at baseline, day 14, day 21, day 28 and at 36 weeks CGA

• Cranial ultrasound and thyroid ultrasound will be measured at 36 weeks CGA

• Magnetic resonance imaging (MRI) and magnetic resonance angiography (MRA) scan at term equivalent

### Design of MRI study

MRI and MRA will be performed at a 1.5 Tesla Achieva Nova MRI scanners (Phillips Medical Systems, Best, Netherlands) at the Royal Liverpool Children's Hospital and the Wellcome Trust Clinical Research Facility (Manchester). A phased array SENSE head coil will be used to facilitate the production of high quality MR images with high signal-to-noise ratio (SNR).

The total scanning time is approximated at 30 minutes and the protocol sequence is detailed in Table 2. Sequencing is prioritized in the order listed and will depend on how long each infant will remain still in the scanner. Data acquisition is not expected to exceed 40 minutes.

**Table 2:** Sequence Protocol for TIPIT

**Sequence**	**Scan Parameters**	**Target area**
1. Midline sagittal T1 weighted Turbo Spin Echo (TSE)	TR = 337 ms, TE = 18 ms Slice thickness = 3 mm, 0.3 mm gap Scan Duration = 2 minutes	Anatomical alignment and measurement of pituitary height
2. Coronal heavily T2 weighted TSE (whole brain)	TR = 5750 ms, TE = 150 ms Slice thickness = 1.5 mm, contiguous Scan Duration = 5 minutes	Measurement of whole brain volume (cerebrum/cerebellum) and measurement of specific brain structures using stereology:
		• caudate
		• hippocampus
		• thalamus
		• prefrontal cortex
		• inferior frontal gyrus
		• temporal lobe
3. MR Angiography	TR = 25 ms, TE = 6.9 ms Slice thickness = 0.6 mm, 3D Time of Flight (TOF) Multiple Overlapping Thin Slab Acquisition (MOTSA) with a SENSE factor of 2.00. Isotropic resolution = 0.6 × 06 × 06 mm^3 ^Scan Duration = 6 minutes	Blood vessel diameter, tortuosity, and density
4. Diffusion tensor imaging	2D single shot spin-echo Echo-Planar Imaging (SE-EPI) with SENSE factor of 2.5. 24 contiguous axial slices of 3 mm thickness with in-plane isotropic resolution of 2 mm. TR/TE = 3350/74 ms. 32 gradient directions with a b-factor of 700 s/mm2. 2 averages. Scan Duration = 3 minutes 47 s.	Measurement of degree of mean diffusivity and fractional anisotrophy (as markers of water content and myelination) in
		• posterior limb of internal capsule
		• centrum semiovale
		• anterior and posterior corpus callosum
		• frontal white matter
		• occipital white matter

5. Axial T2-weighted TSE	TR = 3023 ms, TE = 150 ms. Axial slices of 3 mm thickness with an in-plane isotropic resolution of 0.7 mm Scan Duration = 3 minutes	In same slice location as DTI to aid region drawing

MRI examination will be performed at term equivalence in infants for whom separate informed parental consent will be obtained. No sedation will be used, as infants will be imaged during natural sleep after having been fed. Careful infant positioning by the radiographer will ensure their comfort while being scanned. Patient comfort during an MRI examination is vital for high image quality, especially when scanning neonates. Supervision will be provided for the length of the MRI scan. Ear protection equipment for this study will comprise of pneumatic headphones and ear plugs using President dental putty[40] to decrease acoustic noise and to ensure infants remain still during and in between the scans. This will help prevent any image degradation from motion artefacts. Additional scanner-inbuilt acoustic noise reduction techniques will be employed such as SOFTONE. This will be especially important as some of the advanced MRI methods (diffusion, angiography) can be noisy and may wake infants up[41].

Clinical reports of the MRI scan will be sent to the named clinician and parents will be informed of the outcome of the scans. Image interpretation will be performed by Consultant Radiologists based at Liverpool and Manchester respectively who will have specialist pediatric MR training.

Anonymised images will be safely archived using 5.2 MB Magneto-Optical-Discs (SONY) that will be purchased specifically for this study. This will enable us to retrieve those images for future reference for a larger scale study.

### Image Analysis

Image analysis will be performed using specialised medical imaging software for structure-specific brain volume measurements (Easymeasure) and software for vessel analysis measurement (ImageJ) provided by the Magnetic Resonance Imaging and Analysis Research Center (MARIARC) and the School of Health Sciences of the University of Liverpool.

Brain structure volumes will be estimated using an established, and mathematically unbiased, stereological technique which employs the Cavalieri methods in conjunction with point counting [42-44]. Efficient sampling strategies for estimating the volumes of the structures listed in Table 2 have already been developed and employed in numerous clinical studies[43,45-50].

DTI analysis will use DTIstudio to produce maps of mean diffusivity and fractional anisotropy[38,39]. Regions of interest in the structures listed in Table 2 will be drawn by hand and the mean values recorded. In addition, fibre tracts originating from these regions of interest will be computed, thresholded at fractional anisotropy > 0.15. The number and mean length of fibres will be recorded and also the mean fractional anisotropy and mean diffusivity in the tracked pathways. Further analysis will consider the use of voxel-based morphometry to identify regional group differences in fractional anisotropy and mean diffusivity without a priori information[51]. Two identical phantoms containing n-tridecane will be scanned regularly on both the Manchester and Alder Hey scanners to monitor the accuracy of the mean diffusivity measurements[52].

Vessel diameter will be quantified using ImageJ software and the Full-Width-at-Half-Maximum (FWHM) criterion, as suggested by previous studies[53]. The diameters of the proximal branches of the MCA (M1 segment) and PCA (P1 segment) for both the left and the right side will be measured in the transverse plane of the MIP image.

Tortuosity will be quantified using the distance factor (DF) defined by the following ratio[54]:

$$DF=\frac{\text{Standardised vessel length between defined end points}}{\text{Length of the straight line with same end points}}$$

Analysis will be performed by manually tracing along vessels using the segmented line function of the medical image analysis software ImageJ. A standardised anatomical length of 3 cm starting from the origin of the vessels at the ICA (for the ACA and MCA) or at the basilar artery (for the PCA) will be used.

Vessel density will be qualitatively assessed on the maximum intensity projection images (MIPs) on all three orthogonal planes (axial, sagittal and coronal) and classified in five different categories, ranging from 1 (no vessel visible) to 5 (very dense vasculature)[55].

### Data Management

Data will be collected on case report forms (CRF) and then entered on to a MACRO database. Data will be entered blind to treatment allocation on to the password-protected database. Access to the database will be restricted to the investigators. All CRF hard copies will be stored in a locked filing cabinet within the recruiting centres.

### Statistical Analysis

#### Sample Size Calculation

The power calculation for the sample size of the study was based on a sample size of 64 in each group which will have 80% power to detect a difference between the means of the thyroxine and placebo groups of 0.67 mm (0.5*SD) for the outcome subarachnoid space at 36 weeks' CGA[37]. This assumes that the common standard deviation is 1.3 mm and analysis is based on using a two group t-test with a 0.05 two-sided significance level. The value for the SD is taken from Armstrong et al[56] who report subarachnoid space as an indirect method of brain growth in preterm infants.

#### Data Analyses

Primary analysis of the data will be by intention to treat. Differences between TIPIT participants consenting to the MRI scan and those not consenting will be investigated.

A supplementary analysis will be per protocol for the TIPIT study. This will allow us to assess how thyroid hormone status is associated with brain development.

The volumes of key brain structures, whole brain volume, extent of myelination of cerebral white matter and white matter integrity and brain vessel morphology will be calculated for each baby and difference between the two intervention groups will be compared between the two groups using a two group t-test. The difference in means will be presented with a 95% confidence interval. Multiple regression will be used to adjust for baseline imbalance between the groups and for potential prognostic factors which will be identified prior to the analysis.

### Ethical and Regulatory Issues

#### Patient Consent and Data Monitoring Committee

Parents/guardian will be given full verbal and written information regarding the trial before written consent is obtained. An independent Data Monitoring and Ethics Committee (DMEC) will be formed. During the period of recruitment, interim summaries of results will be supplied, in strictest confidence, to the DMEC by the trial statistician (see main TIPIT protocol[37] for details).

#### Regulatory bodies

EUDRACT number : 2005-003-09939

MHRA approval was granted 15^th ^June 2007

North West Research Ethics Committee gave a favourable ethical opinion to the study on 12^th ^June 2007.

#### Funders

Medical Research Council and the Newborn Appeal

## Competing interests

The authors declare that they have no competing interests.

## Authors' contributions

SMN conceived the TIPIT/cranial MRI study, participated in the design and management of the TIPIT trial, the design and coordination of the cranial MRI study and drafted the manuscript. AMW and MAT conceived the TIPIT/cranial MRI study, and participated in its design and management of the TIPIT trial. MD and SV participated in the design of the TIPIT trial and its management and the design and coordination of the cranial MRI study. CG participated in the design of the study and the statistical analysis. LMP, VS and LA conceived the cranial MRI study and participated in its design and coordination. AT, CM and LG participated in design sequence on the MRI protocol. All authors read and approved the final manuscript.

## Pre-publication history

The pre-publication history for this paper can be accessed here:



## References

[B1] Wood NS, Costeloe K, Gibson AT, Hennessy EM, Marlow N, Wilkinson AR (2005). The EPICure study: associations and antecedents of neurological and developmental disability at 30 months of age following extremely preterm birth. Arch Dis Child Fetal Neonatal Ed.

[B2] Bourgeois JP (1997). Synaptogenesis, heterochrony and epigenesis in the mammalian neocortex. Acta Paediatr Suppl.

[B3] Abernethy LJ, Cooke RW, Foulder-Hughes L (2004). Caudate and hippocampal volumes, intelligence, and motor impairment in 7-year-old children who were born preterm. Pediatr Res.

[B4] Abernethy LJ, Klafkowski G, Foulder-Hughes L, Cooke RW (2003). Magnetic resonance imaging and T2 relaxometry of cerebral white matter and hippocampus in children born preterm. Pediatr Res.

[B5] Inder TE, Huppi PS, Warfield S, Kikinis R, Zientara GP, Barnes PD, Jolesz F, Volpe JJ (1999). Periventricular white matter injury in the premature infant is followed by reduced cerebral cortical gray matter volume at term. Ann Neurol.

[B6] Inder TE, Warfield SK, Wang H, Huppi PS, Volpe JJ (2005). Abnormal cerebral structure is present at term in premature infants. Pediatrics.

[B7] Wood NS, Costeloe K, Gibson AT, Hennessy EM, Marlow N, Wilkinson AR, Wood NS, Marlow N, Costeloe K, Gibson AT, Wilkinson AR (2003). The EPICure study: growth and associated problems in children born at 25 weeks of gestational age or less
Neurologic and developmental disability after extremely preterm birth. EPICure Study Group. Arch Dis Child Fetal Neonatal Ed.

[B8] Marlow N, Wolke D, Bracewell MA, Samara M (2005). Neurologic and developmental disability at six years of age after extremely preterm birth. N Engl J Med.

[B9] Marlow N (2004). Neurocognitive outcome after very preterm birth. Arch Dis Child Fetal Neonatal Ed.

[B10] Cooke RW (2006). Are there critical periods for brain growth in children born preterm?. Arch Dis Child Fetal Neonatal Ed.

[B11] Cai D, Su Q, Chen Y, Luo M (2000). Effect of thyroid hormone deficiency on developmental expression of goalpha gene in the brain of neonatal rats by competitive RT-PCR and in situ hybridization histochemistry. Brain Res.

[B12] Bernal J (2007). Thyroid hormone receptors in brain development and function. Nat Clin Pract Endocrinol Metab.

[B13] Iniguez MA, De Lecea L, Guadano-Ferraz A, Morte B, Gerendasy D, Sutcliffe JG, Bernal J (1996). Cell-specific effects of thyroid hormone on RC3/neurogranin expression in rat brain. Endocrinology.

[B14] Auso E, Lavado-Autric R, Cuevas E, Del Rey FE, Morreale De Escobar G, Berbel P (2004). A moderate and transient deficiency of maternal thyroid function at the beginning of fetal neocorticogenesis alters neuronal migration. Endocrinology.

[B15] Lavado-Autric R, Auso E, Garcia-Velasco JV, Arufe Mdel C, Escobar del Rey F, Berbel P, Morreale de Escobar G (2003). Early maternal hypothyroxinemia alters histogenesis and cerebral cortex cytoarchitecture of the progeny. J Clin Invest.

[B16] Eayrs JT, Horn G (1955). The development of cerebral cortex in hypothyroid and starved rats. Anat Rec.

[B17] Eayrs JT (1953). Thyroid hypofunction and the development of the central nervous system. Nature.

[B18] Berbel P, Auso E, Garcia-Velasco JV, Molina ML, Camacho M (2001). Role of thyroid hormones in the maturation and organisation of rat barrel cortex. Neuroscience.

[B19] Berbel P, Guadano-Ferraz A, Angulo A, Ramon Cerezo J (1994). Role of thyroid hormones in the maturation of interhemispheric connections in rats. Behav Brain Res.

[B20] Nicholson JL, Altman J (1972). The effects of early hypo- and hyperthyroidism on the development of the rat cerebellar cortex. II. Synaptogenesis in the molecular layer. Brain Res.

[B21] Xiao Q, Nikodem VM (1998). Apoptosis in the developing cerebellum of the thyroid hormone deficient rat. Fron Biosci.

[B22] Balazs R, Kovacs S, Cocks WA, Johnson AL, Eayrs JT (1971). Effect of thyroid hormone on the biochemical maturation of rat brain: postnatal cell formation. Brain Res.

[B23] Balazs R, Brooksbank BW, Davison AN, Eayrs JT, Wilson DA (1969). The effect of neonatal thyroidectomy on myelination in the rat brain. Brain Res.

[B24] Heuer H (2007). The importance of thyroid hormone transporters for brain development and function. Best Practice and Research Clinical Endocrinology and Metabolism.

[B25] Gupta RK, Bhatia V, Poptani H, Gujral RB (1995). Brain metabolite changes on in vivo proton magnetic resonance spectroscopy in children with congenital hypothyroidism. J Pediatr.

[B26] Jagannathan NR, Tandon N, Raghunathan P, Kochupillai N (1998). Reversal of abnormalities of myelination by thyroxine therapy in congenital hypothyroidism: localized in vivo proton magnetic resonance spectroscopy (MRS) study. Brain Res Dev Brain Res.

[B27] Crisanti P OB (2001). The expression of thyrotropin receptors in the brain.. Endocrinology.

[B28] Vohr BR, Allen M (2005). Extreme prematurity--the continuing dilemma. N Engl J Med.

[B29] Chowdhry P, Scanlon JW, Auerbach R, Abbassi V (1984). Results of controlled double-blind study of thyroid replacement in very low-birth-weight premature infants with hypothyroxinemia. Pediatrics.

[B30] Williams FL, Simpson J, Delahunty C, Ogston SA, Bongers-Schokking JJ, Murphy N, van Toor H, Wu SY, Visser TJ, Hume R (2004). Developmental trends in cord and postpartum serum thyroid hormones in preterm infants. J Clin Endocrinol Metab.

[B31] van Wassenaer AG, Kok JH, Dekker FW, de Vijlder JJ (1997). Thyroid function in very preterm infants: influences of gestational age and disease. Pediatr Res.

[B32] Biswas S, Buffery J, Enoch H, Bland JM, Walters D, Markiewicz M (2002). A longitudinal assessment of thyroid hormone concentrations in preterm infants younger than 30 weeks' gestation during the first 2 weeks of life and their relationship to outcome. Pediatrics.

[B33] Fisher DA, Klein AH (1981). Thyroid development and disorders of thyroid function in the newborn. N Engl J Med.

[B34] van Wassenaer AG, Kok JH, de Vijlder JJ, Briet JM, Smit BJ, Tamminga P, van Baar A, Dekker FW, Vulsma T (1997). Effects of thyroxine supplementation on neurologic development in infants born at less than 30 weeks' gestation. N Engl J Med.

[B35] van Wassenaer AG, Westera J, B.A. H, Kok JH (2005). Ten year follow up of children born at < 30 weeks' gestational age supplemented with thyroxine in the neonatal period in a randomized controlled trial. Pediatrics.

[B36] Leviton A, Paneth N, Reuss ML, Susser M, Allred EN, Dammann O, Kuban K, Van Marter LJ, Pagano M (1999). Hypothyroxinemia of prematurity and the risk of cerebral white matter damage. J Pediatr.

[B37] Ng SM, Turner MA, Gamble C, Didi M, Victor S, Weindling AM (2008). TIPIT: A randomised controlled trial of thyroxine in preterm infants under 28 weeks' gestation. Trials.

[B38] Nair G, Tanahashi Y, Low HP, Billings-Gagliardi S, Schwartz WJ, Duong TQ (2005). Myelination and long diffusion times alter diffusion-tensor-imaging contrast in myelin-deficient shiverer mice. Neuroimage.

[B39] Jiang H, van Zijl PC, Kim J, Pearlson GD, Mori S (2006). DtiStudio: resource program for diffusion tensor computation and fiber bundle tracking. Comput Methods Programs Biomed.

[B40] O'Shea TM, Counsell SJ, Bartels DB, Dammann O (2005). Magnetic resonance and ultrasound brain imaging in preterm infants. Early Hum Dev.

[B41] Malamateniou C, Counsell SJ, Allsop JM, Fitzpatrick JA, Srinivasan L, Cowan FM, Hajnal JV, Rutherford MA (2006). The effect of preterm birth on neonatal cerebral vasculature studied with magnetic resonance angiography at 3 Tesla. Neuroimage.

[B42] Roberts N, Puddephat MJ, McNulty V (2000). The benefit of stereology for quantitative radiology. Br J Radiol.

[B43] Garcia-Finana M, Cruz-Orive LM, Mackay CE, Pakkenberg B, Roberts N (2003). Comparison of MR imaging against physical sectioning to estimate the volume of human cerebral compartments. Neuroimage.

[B44] Garcia-Finana M (2006). Confidence intervals in Cavalieri sampling. J Microsc.

[B45] Keller SS, Highley JR, Garcia-Finana M, Sluming V, Rezaie R, Roberts N (2007). Sulcal variability, stereological measurement and asymmetry of Broca's area on MR images. J Anat.

[B46] Gong QY, Sluming V, Mayes A, Keller S, Barrick T, Cezayirli E, Roberts N (2005). Voxel-based morphometry and stereology provide convergent evidence of the importance of medial prefrontal cortex for fluid intelligence in healthy adults. Neuroimage.

[B47] Howard MA, Roberts N, Garcia-Finana M, Cowell PE (2003). Volume estimation of prefrontal cortical subfields using MRI and stereology. Brain Res Brain Res Protoc.

[B48] Mackay CE, Webb JA, Eldridge PR, Chadwick DW, Whitehouse GH, Roberts N (2000). Quantitative magnetic resonance imaging in consecutive patients evaluated for surgical treatment of temporal lobe epilepsy. Magn Reson Imaging.

[B49] Cowell PE, Sluming VA, Wilkinson ID, Cezayirli E, Romanowski CA, Webb JA, Keller SS, Mayes A, Roberts N (2007). Effects of sex and age on regional prefrontal brain volume in two human cohorts. Eur J Neurosci.

[B50] Howard MA, Cowell PE, Boucher J, Broks P, Mayes A, Farrant A, Roberts N (2000). Convergent neuroanatomical and behavioural evidence of an amygdala hypothesis of autism. Neuroreport.

[B51] Ashburner J, Friston KJ (2000). Voxel-based morphometry--the methods. Neuroimage.

[B52] Tofts PS, Lloyd D, Clark CA, Barker GJ, Parker GJ, McConville P, Baldock C, Pope JM (2000). Test liquids for quantitative MRI measurements of self-diffusion coefficient in vivo. Magn Reson Med.

[B53] Hoogeveen RM, Bakker CJ, Viergever MA (1998). Limits to the accuracy of vessel diameter measurement in MR angiography. J Magn Reson Imaging.

[B54] Bullitt E, Gerig G, Pizer SM, Lin W, Aylward SR (2003). Measuring tortuosity of the intracerebral vasculature from MRA images. IEEE Trans Med Imaging.

[B55] Malamateniou C, Counsell SJ, Allsop JM (2005). Optimised magnetic resonance angiography at 3 Tesla for neonates.

[B56] Armstrong DL, Bagnall C, Harding JE, Teele RL (2002). Measurement of the subarachnoid space by ultrasound in preterm infants. Arch Dis Child Fetal Neonatal Ed.

[B57] Reuss ML, Paneth N, Pinto-Martin JA, Lorenz JM, Susser M (1996). The relation of transient hypothyroxinemia in preterm infants to neurologic development at two years of age. N Engl J Med.

[B58] Den Ouden AL, Kok JH, Verkerk PH, Brand R, Verloove-Vanhorick SP (1996). The relation between neonatal thyroxine levels and neurodevelopmental outcome at age 5 and 9 years in a national cohort of very preterm and/or very low birth weight infants. Pediatr Res.

[B59] Paul DA, Leef KH, Stefano JL, Bartoshesky L (1998). Low serum thyroxine on initial newborn screening is associated with intraventricular hemorrhage and death in very low birth weight infants. Pediatrics.

[B60] Lucas A, Morley R, Fewtrell MS (1996). Low triiodothyronine concentration in preterm infants and subsequent intelligence quotient (IQ) at 8 year follow up. Bmj.

[B61] Meijer WJ, Verloove-Vanhorick SP, Brand R, van den Brande JL (1992). Transient hypothyroxinaemia associated with developmental delay in very preterm infants. Arch Dis Child.

